# Association between Socioeconomic Factors and Cancer Risk: A Population Cohort Study in Scotland (1991-2006)

**DOI:** 10.1371/journal.pone.0089513

**Published:** 2014-02-27

**Authors:** Katharine H. Sharpe, Alex D. McMahon, Gillian M. Raab, David H. Brewster, David I. Conway

**Affiliations:** 1 Information Services Division, NHS National Services Scotland, Edinburgh, Scotland, United Kingdom; 2 University of Glasgow, College of Medical, Veterinary and Life Sciences: Dental School, Glasgow, Scotland, United Kingdom; 3 University of St Andrews, St Andrews, Fife, Scotland, United Kingdom; 4 Public Health Sciences, Edinburgh University Medical School, Edinburgh, Scotland, United Kingdom; Universität Bochum, Germany

## Abstract

**Background:**

Lung and upper aero-digestive tract (UADT) cancer risk are associated with low socioeconomic circumstances and routinely measured using area socioeconomic indices. We investigated effect of country of birth, marital status, one area deprivation measure and individual socioeconomic variables (economic activity, education, occupational social class, car ownership, household tenure) on risk associated with lung, UADT and all cancer combined (excluding non melanoma skin cancer).

**Methods:**

We linked Scottish Longitudinal Study and Scottish Cancer Registry to follow 203,658 cohort members aged 15+ years from 1991–2006. Relative risks (RR) were calculated using Poisson regression models by sex offset for person-years of follow-up.

**Results:**

21,832 first primary tumours (including 3,505 lung, 1,206 UADT) were diagnosed. Regardless of cancer, economically inactivity (versus activity) was associated with increased risk (male: RR 1.14, 95% CI 1.10–1.18; female: RR 1.06, 95% CI 1.02–1.11). For lung cancer, area deprivation remained significant after full adjustment suggesting the area deprivation cannot be fully explained by individual variables. No or non degree qualification (versus degree) was associated with increased lung risk; likewise for UADT risk (females only). Occupational social class associations were most pronounced and elevated for UADT risk. No car access (versus ownership) was associated with increased risk (excluding all cancer risk, males). Renting (versus home ownership) was associated with increased lung cancer risk, UADT cancer risk (males only) and all cancer risk (females only). Regardless of cancer group, elevated risk was associated with no education and living in deprived areas.

**Conclusions:**

Different and independent socioeconomic variables are inversely associated with different cancer risks in both sexes; no one socioeconomic variable captures all aspects of socioeconomic circumstances or life course. Association of multiple socioeconomic variables is likely to reflect the complexity and multifaceted nature of deprivation as well as the various roles of these dimensions over the life course.

## Background

The association of socioeconomic status (SES) and health is well established and shows a mostly consistent pattern of poorer health with lower SES [1]; [2]. SES is usually measured in routine statistics using an area indicator or in epidemiological studies with a single indicator such as education. Lung and upper aero-digestive tract (UADT) cancers taken together are the most common cancers in the world compared to the other individual sites; 21% of global cases were diagnosed in Europe in 2008 [3]. These cancers show stark socioeconomic inequalities with greater incidence among lower socioeconomic groups [4]–[7]. The United Kingdom (UK) has the second highest age standardised incidence rate (ASR) for these cancers among Northern European countries with Scotland ranking the highest in the UK [3]; [8]. In Scotland, cancer incidence is higher in more deprived areas with the level of inequality remaining stable over time [9]. Furthermore, lung and UADT cancers contributed 90% (males) and 81% (females) to total social inequality in cancer risk in Scotland when measured using the recently developed Scottish Index of Multiple Deprivation, an area measure of social circumstances [10].

While the relative importance of area and individual SES association with cancer mortality has been explored, more limited work has focused on cancer incidence [11]; [12]. Such studies have more frequently focused on single SES factors such as occupational social class [13]–[15], highest education level attained [16], or disposable income [16]. Others have explored an individual SES measure such as education level and area characteristics in terms of attributes such as rural versus urban status [17] or European region [18] while many have studied area SES variables alone [11]; [18]. Other social indicators including marital status have been associated with increased cancer risk [19]. Moreover, all cancer, lung, colorectal, breast and prostate cancer mortality by country of birth showed higher mortality for all cancer and lung cancer among people born in Scotland [20]; [21]. Few studies have assessed the association with cancer incidence of both area and individual SES variables along with marriage status and country of birth [22]; [23].

Here, we explored the association of cancer risk with one demographic variable (country of birth), one social variable (marital status), one area SES variable through Carstairs deprivation index [24] and five individual socioeconomic variables (economic activity, occupational social class, education level, car ownership, and household tenure). We aimed to reassess more finely the socioeconomic factors associated with cancer risk through: (i) examining the consistency of relationship between an area and several individual SES measures and cancer risk; (ii) establishing if any single measure was particularly associated with cancer incidence; (iii) assessing if the area measure was fully explained by the individual measures; and (iv) exploring if there were any synergistic effects between the area deprivation measure and each individual SES variable.

## Methods

We linked 1991 Census data and mortality data from the Scottish Longitudinal Study (SLS) [25] managed by National Records of Scotland (NRS) to data from the Scottish Cancer Registry (SCR) managed by NHS National Services Scotland (NSS) to develop a cohort. The SLS links data from the Censuses and other administrative sources for a semi-random 5.3% representative sample of the Scottish population. It is the only administrative source of self-reported individual SES factors in Scotland. We chose the five individual categorical socioeconomic variables from the 1991 Census based on the variable's ability to capture SES at various stages of life and the variable's focus on established and different determinants of SES [26]–[29]. We also included country of birth (Scotland, rest of UK, rest of world) and legal marriage status (single, married, widowed, and divorced).

Economic activity was grouped into active (full time and part time employees, self-employed, on a government scheme) and inactive (waiting to start a job, unemployed, student status, permanently sick, retired, looking after home or family, or other inactive). Occupational social class was grouped using the Registrar General defined categories: Social Class I (professional, managerial, technical), Social Class II (intermediate), Social Class, IIINM (skilled non-manual), Social Class IIIM (skilled manual), Social Class, IV (partly skilled), and Social Class V (unskilled) [30]. Education qualifications reflected highest attained degree (first degree and higher, other non degree, none or missing or under 18 years old). Car ownership was grouped into one or more cars or no car, while household tenure was grouped into owned (owner occupier) or rented (with job, farm or other business, local authority or council, new town corporation, housing association or charitable trust, or private landlord). All variables were measured at 1991 Census, the start of the follow up period.

We used Carstairs decile as the area deprivation measure providing the socioeconomic environmental dimension. Carstairs is measured for Scotland's 1,011 postcode sectors with average population 5,012 and is based on the area level measure of four decennial census variables here taken from the 1991 Census: male unemployment, households with no car, overcrowded households, and the percentage of people in higher occupational socioeconomic classes. Unlike other more recent area measures, Carstairs was available for 1991, the start of our cohort [31].

The study population consisted of 206,830 SLS members 15+ years old present at the 1991 Census and who had been traced at the NHS Central Register so that follow up data were available. These records linked to individual SCR records recording date of diagnosis and diagnosis code for first primary cancers. 2,950 individuals diagnosed with cancer prior to 1 April 1991 and 222 individuals with a missing Carstairs score were excluded leaving 203,658 cohort members who were followed for up to 16 years from the study start (the 1991 Census date) to the study end date defined as the earliest date of incident cancer, death or the 31 December 2006.

We analysed first primary incident cancers excluding non melanoma skin cancer (here after referred to as all cancer (C00-C96, excluding C44) lung cancer (C33, C34) and upper aero digestive tract (UADT) cancers (C00 – C14, C30-C32, C15).

The relative risks (RR) and 95% confidence intervals (CI) were computed using Poisson regression models by sex corrected for under dispersion and offset by person-years of follow-up adjusted for age at start of the cohort in 10 year categories beginning with 45–54 years (minimally adjusted model). We also established the RR of cancer for each variable category by mutually adjusting all the variables for each other (fully adjusted model). Reference categories used for each variable were: country of birth (Scotland), marital status (married), area SES (least deprived), economic activity (active), education level (first degree and higher), occupational social class (professional, managerial, technical), car ownership (1 or more car(s)), and household tenure (owned). RRs with 95% CI that did not include the value of 1.0 were regarded as statistically significant. We also tested for the relationship between area deprivation and education level in a stratification analysis. Finally, using the multivariate Poisson models, we tested the interaction between area deprivation and each individual socioeconomic variable as well as the difference in RRs between the sexes (females as reference); significance was established at P<0.0001. We conducted age adjusted sub group analyses to explore further statistically significant area and individual socioeconomic variable interactions. All analyses were performed using SAS version 9.2 (SAS Institute Inc. USA).

The University of Glasgow Medical Ethics Committee, NSS Privacy Advisory Committee and SLS Research Board approved this study. Analysis was conducted on a secure standalone computer, following strict disclosure protocols. Outputs leaving the safe setting (including this paper) were screened for disclosure by SLS prior to release. Data are publically available to researchers through a similar process of approvals and access.

## Results

The cohort consisted of 203,658 individuals (106,819 females and 96,839 males) present in the 1991 Census with an average age of 42.8 years (Table 1). 21,832 first primary cancers were diagnosed during 3.05 million person-years of follow-up (52.3% male, 47.7% female). 3,505 lung cancer cases were diagnosed during 3.12 million person-years of follow-up (52.6% female, 47.4% male) and 1,206 UADT cancer cases during 3.12 million person-years of follow-up (52.6%. female, 47.4% male) (Tables 2 and 3).

**Table 1:** Cohort number, proportion, average (avg) age and standard deviation (SD) by variable and sex, April 1991, Scotland.

		a) Females				b) Males			
				Age				Age	
		Number	(%)	Avg	SD	Number	(%)	Avg	SD
Total		106,819	(100.0)	45.2	19.4	96,839	(100.0)	42.8	17.9
Country of birth	Scotland	95,057	(88.9)	45.3	19.5	85,802	(88.6)	42.7	18.0
	Rest UK	8,710	(8.2)	44.8	18.9	8,259	(8.5)	43.4	17.3
	Rest of World	3,052	(2.9)	44.6	18.6	2,778	(2.9)	44.6	18.0
Marital Status	Married	60,425	(56.6)	46.0	14.7	59,920	(61.9)	48.2	14.9
	Divorced	5,832	(5.5)	44.2	13.0	4,163	(4.3)	45.1	12.1
	Single	26,610	(24.9)	30.2	18.4	29,120	(30.1)	27.8	13.9
	Widowed	13,952	(13.1)	71.3	11.1	3,636	(3.8)	70.6	11.4
Carstairs area	1 Least Deprived	8,698	(8.1)	44.2	18.1	8,411	(8.7)	42.8	17.1
	2	10,007	(9.4)	44.6	18.9	9,504	(9.8)	42.3	17.1
	3	12,897	(12.1)	45.1	19.0	11,906	(12.3)	42.9	17.5
	4	13,131	(12.3)	45.5	19.3	12,344	(12.7)	42.8	18.0
	5	11,995	(11.2)	45.7	19.7	10,854	(11.2)	43.0	18.1
	6	11,487	(10.8)	46.5	20.0	10,068	(10.4)	43.4	18.5
	7	9,963	(9.3)	46.2	19.7	8,872	(9.2)	43.5	18.4
	8	9,988	(9.4)	45.3	19.9	8,964	(9.3)	43.1	18.1
	9	9,216	(8.6)	45.3	19.7	7,995	(8.3)	42.6	18.4
	10 Most deprived	9,437	(8.8)	43.4	19.5	7,921	(8.2)	41.4	18.0
Economic activity	Economically active	53,249	(50.6)	36.8	12.6	70,719	(73)	38.1	13.1
	Economically inactive	51,958	(49.4)	54.9	20.4	24,452	(25.3)	58.5	20.3
	Under 16 years old	1,612	(1.5)	15.0	0.0	1,668	(1.7)	15.0	0.0
Education level	First degree and higher	4,823	(5)	38.7	14.6	7,066	( 8.0)	41.1	14.2
	Other non degree	8,653	(8.9)	43.2	15.2	6,404	(7.3)	43.4	14.8
	None	83,421	(86.1)	47.2	19.0	74,757	(84.7)	44.6	17.5
	Under 18 years old or missing^1^	9,922	(10.2)	33.5	22.5	8,612	(9.8)	28.2	19.6
Occupational social class	I, II Professional, managerial, technical	18,454	(17.3)	40.2	13.1	23,434	(24.2)	43.2	13.7
	III N Skilled non manual	25,462	(5.1)	37.2	14.5	9,347	(9.7)	38.0	15.6
	III M Skilled manual	5,481	(23.8)	37.7	15.0	26,577	(27.4)	40.8	14.9
	IV Partly skilled	11,579	(10.8)	37.6	14.5	14,359	(14.8)	40.6	16.2
	VI Unskilled	7,252	(6.8)	46.6	13.7	4,609	(4.8)	39.8	16.1
	No job in last 10 years, under 16 years old or missing^2^	38,591	(36.1)	56.1	22.3	18,513	(19.1)	50.1	25.7
Car ownership	1 or more car(s)	66,422	(62.2)	41.2	16.6	68,702	(70.9)	41.2	16.6
	No cars	40,397	(37.8)	51.8	21.7	28,137	(29.1)	46.7	20.2
Household tenure	Owned	59,032	(55.3)	43.7	18.2	56,760	(58.6)	42.0	17.0
	Rented	47,787	(44.7)	47.1	20.7	40,079	(41.4)	43.9	19.1

1 5.04% of total population was under 18 years old therefore education not recorded; 4.1% of total population education level not stated.

2 For 0.4% of total population occupational social class was not adequately described or not stated, 27.5% of total population was less than 16 years old or held no job in last 10 years.

Source: Scottish Longitudinal Study.

**Table 2:** Age adjusted relative risks (RR) and 95% confidence intervals (CI) by cancer, demographic or socioeconomic variable for females, Scotland 1991–2006.

		All cancer				Lung				UADT			
	Level	Number of cases	RR	95% CI		Number of cases	RR	95% CI		Number of cases	RR	95% CI	
Country of birth	Scotland	10946	Reference			1344	Reference			416	Reference		
	Rest UK	869	**0.87**	**0.83**	**0.92**	66	**0.55**	**0.51**	**0.60**	30	**0.78**	**0.72**	**0.85**
	Rest of World	254	**0.73**	**0.66**	**0.81**	20	**0.47**	**0.41**	**0.55**	7	**0.53**	**0.45**	**0.62**
Marital status	Married	6721	Reference			767	Reference			235	Reference		
	Divorced	696	**1.19**	**1.12**	**1.26**	92	**1.50**	**1.39**	**1.61**	34	**1.83**	**1.70**	**1.98**
	Single	1979	**0.92**	**0.89**	**0.96**	94	**0.64**	**0.59**	**0.69**	51	**0.91**	**0.85**	**0.98**
	Widowed	2673	**1.11**	**1.06**	**1.15**	477	**1.50**	**1.44**	**1.57**	133	**1.18**	**1.12**	**1.24**
Area deprivation	1 Least Deprived	899	Reference			65	Reference			31	Reference		
	2	993	0.95	0.88	1.02	88	**1.16**	**1.04**	**1.30**	34	**0.88**	**0.79**	**0.97**
	3	1359	0.99	0.92	1.05	119	**1.17**	**1.06**	**1.30**	49	0.97	0.88	1.07
	4	1502	1.07	1.00	1.14	155	**1.49**	**1.35**	**1.64**	46	0.90	0.82	1.00
	5	1324	1.02	0.95	1.09	158	**1.63**	**1.47**	**1.79**	37	**0.75**	**0.67**	**0.83**
	6	1347	1.05	0.98	1.12	151	**1.54**	**1.40**	**1.71**	56	**1.13**	**1.03**	**1.24**
	7	1217	**1.09**	**1.02**	**1.17**	147	**1.72**	**1.55**	**1.90**	48	**1.18**	**1.07**	**1.30**
	8	1204	**1.11**	**1.04**	**1.19**	155	**1.91**	**1.73**	**2.11**	49	**1.21**	**1.10**	**1.34**
	9	1100	**1.10**	**1.02**	**1.18**	195	**2.59**	**2.36**	**2.85**	52	**1.37**	**1.24**	**1.50**
	10 Most deprived	1124	**1.15**	**1.07**	**1.23**	197	**2.80**	**2.55**	**3.09**	51	**1.45**	**1.32**	**1.60**
Economic activity	Economically active	4126	Reference			310	Reference			109	Reference		
	Economically inactive	7864	**1.07**	**1.03**	**1.11**	1120	**1.47**	**1.40**	**1.54**	344	**1.31**	**1.24**	**1.38**
	Under 16 years old	79	**0.82**	**0.69**	**0.98**	0	0.00	0.00	0.00	0	0.00	0.00	0.00
Education level	First degree and higher	386	Reference			12	Reference			7	Reference		
	Other non degree	806	0.99	0.90	1.09	56	**1.78**	**1.44**	**2.20**	25	**1.52**	**1.26**	**1.82**
	None	9979	**1.10**	**1.02**	**1.19**	1280	**3.24**	**2.67**	**3.94**	394	**1.88**	**1.59**	**2.22**
	Under 18 years old or missing^1^	898	1.02	0.93	1.12	82	**2.69**	**2.18**	**3.31**	27	**1.53**	**1.27**	**1.84**
Occupational social class	I, II Professional, managerial, technical	1638	Reference			120	Reference			41	Reference		
	IIIa N Skilled non manual	2250	1.03	0.98	1.09	157	**1.04**	**0.95**	**1.12**	63	**1.16**	**1.07**	**1.26**
	IIIb M Skilled manual	525	**1.09**	**1.01**	**1.18**	65	**1.88**	**1.69**	**2.08**	21	**1.68**	**1.50**	**1.89**
	IV Partly skilled	1137	**1.14**	**1.07**	**1.21**	117	**1.66**	**1.52**	**1.81**	38	**1.57**	**1.43**	**1.72**
	V Unskilled	926	**1.14**	**1.07**	**1.22**	173	**2.28**	**2.11**	**2.47**	37	**1.48**	**1.35**	**1.63**
	No job in last 10 years, under 16 years old or missing^2^	5593	**1.07**	**1.02**	**1.12**	798	**1.66**	**1.55**	**1.78**	253	**1.43**	**1.32**	**1.54**
Car ownership	1 or more car(s)	6427	Reference			560	Reference			195	Reference		
	No car	5642	**1.15**	**1.11**	**1.18**	870	**1.82**	**1.75**	**1.89**	258	**1.45**	**1.39**	**1.52**
Household tenure	Owned	5987	Reference			515	Reference			200	Reference		
	Rented	6082	**1.14**	**1.11**	**1.18**	915	**1.90**	**1.83**	**1.97**	253	**1.31**	**1.25**	**1.36**

1 4.6% of total population was under 18 years old therefore education not recorded; 4.6% of total population education level not stated.

2 0.4% of total population occupational social class was not adequately described or not stated, 35.7% of total population was less than 16 years old or held no job in last 10 years.

Source: Scottish Longitudinal Study.

**Table 3:** Age adjusted relative risks (RR) and 95% confidence intervals (CI) by cancer, demographic or socioeconomic variable for males, Scotland 1991–2006.

		All cancer				Lung				UADT			
	Level	Number of cases	RR	95% CI		Number of cases	RR	95% CI		Number of cases	RR	95% CI	
Country of birth	Scotland	8765	Reference			1894	Reference			698	Reference		
	Rest UK	731	**0.87**	**0.82**	**0.91**	125	**0.68**	**0.63**	**0.73**	47	**0.67**	**0.62**	**0.73**
	Rest of World	267	**0.86**	**0.79**	**0.94**	56	**0.84**	**0.75**	**0.93**	8	**0.36**	**0.30**	**0.44**
Marital status	Married	7452	Reference			1558	Reference			552	Reference		
	Divorced	449	**1.11**	**1.04**	**1.19**	123	**1.52**	**1.41**	**1.63**	55	**1.65**	**1.52**	**1.78**
	Single	941	**0.71**	**0.67**	**0.74**	140	**0.67**	**0.62**	**0.72**	92	**0.87**	**0.81**	**0.93**
	Widowed	921	**1.01**	**0.96**	**1.07**	254	**1.30**	**1.23**	**1.37**	54	1.14	1.05	1.23
Area deprivation	1 Least Deprived	790	Reference			92	Reference			46	Reference		
	2	819	0.95	0.89	1.02	130	**1.31**	**1.18**	**1.46**	46	0.93	0.83	1.04
	3	1124	1.01	0.94	1.07	187	**1.44**	**1.30**	**1.59**	90	**1.45**	**1.31**	**1.60**
	4	1183	0.99	0.93	1.06	208	**1.50**	**1.36**	**1.66**	93	**1.45**	**1.31**	**1.60**
	5	1116	1.05	0.98	1.12	229	**1.84**	**1.67**	**2.03**	87	**1.58**	**1.43**	**1.75**
	6	1043	1.02	0.96	1.09	226	**1.89**	**1.71**	**2.08**	71	**1.29**	**1.16**	**1.43**
	7	1002	**1.12**	**1.05**	**1.20**	237	**2.26**	**2.05**	**2.49**	88	**1.88**	**1.70**	**2.08**
	8	974	**1.09**	**1.02**	**1.17**	238	**2.27**	**2.06**	**2.51**	77	**1.63**	**1.47**	**1.80**
	9	874	**1.11**	**1.04**	**1.19**	249	**2.71**	**2.46**	**2.98**	86	**2.05**	**1.85**	**2.27**
	10 Most deprived	838	**1.16**	**1.08**	**1.24**	279	**3.35**	**3.05**	**3.68**	69	**1.72**	**1.55**	**1.91**
Economic activity	Economically active	4069	Reference			671	Reference			335	Reference		
	Economically inactive	5685	**1.14**	**1.10**	**1.18**	1404	**1.68**	**1.61**	**1.76**	418	**1.67**	**1.59**	**1.75**
	Under 16 years old	9	**0.27**	**0.17**	**0.42**	0	**0.00**	**0.00**	**0.00**	0	**0.00**	**0.00**	**0.00**
Education level	First degree and higher	481	Reference			39	Reference			26	Reference		
	Other non degree	534	1.01	0.92	1.10	59	**1.32**	**1.12**	**1.55**	36	**1.17**	**1.02**	**1.35**
	None	8298	**1.17**	**1.09**	**1.24**	1861	**3.05**	**2.68**	**3.47**	660	**1.82**	**1.63**	**2.03**
	Under 18 years old or missing^1^	450	0.95	0.87	1.04	116	**3.24**	**2.80**	**3.75**	31	**1.40**	**1.21**	**1.62**
Occupational social class	I, II Professional, managerial, technical	2001	Reference			287	Reference			120	Reference		
	IIIa N Skilled non manual	696	1.06	0.99	1.12	109	**1.19**	**1.09**	**1.30**	52	**1.42**	**1.30**	**1.56**
	IIIb M Skilled manual	2384	**1.13**	**1.08**	**1.17**	541	**1.81**	**1.71**	**1.92**	227	**1.82**	**1.71**	**1.93**
	IV Partly skilled	1394	**1.14**	**1.09**	**1.20**	291	**1.67**	**1.56**	**1.78**	119	**1.68**	**1.56**	**1.80**
	V Unskilled	421	**1.14**	**1.06**	**1.23**	97	**1.84**	**1.68**	**2.02**	49	**2.27**	**2.07**	**2.49**
	No job in last 10 years, under 16 years old or missing^2^	2867	**1.11**	**1.06**	**1.16**	750	**2.20**	**2.07**	**2.33**	186	**1.84**	**1.72**	**1.98**
Car ownership	1 or more car(s)	6150	Reference			1073	Reference			430	Reference		
	No car	3613	**1.06**	**1.03**	**1.09**	1002	**1.68**	**1.62**	**1.74**	323	**1.67**	**1.60**	**1.74**
Household tenure	Owned	5199	Reference			847	Reference			360	Reference		
	Rented	4564	**1.08**	**1.05**	**1.11**	1228	**1.76**	**1.70**	**1.82**	393	**1.50**	**1.44**	**1.56**

1 5.4% of total population was under 18 years old therefore education not recorded; 3.5% of total population education level not stated.

2 0.5% of total population occupational social class was not adequately described or not stated, 18.5% of total population was less than 16 years old or held no job in last 10 years.

Source: Scottish Longitudinal Study.

When compared to the relevant referent categories and regardless of sex or cancer group, the minimally adjusted models showed elevated cancer risk association for individuals born in Scotland; divorced or widowed; living in more deprived areas; unemployed; with no education; employed in skilled manual, partly skilled or unskilled jobs; with no access to a car or renting a home (Tables 2 and 3). In the fully adjusted models, RRs for each variable were attenuated (some fully) depending on the sex and cancer group; these differences are detailed by each variable below. With the exception of country of birth and single marital status, all statistically significant RRs were greater for males compared to females (P<0.0001, data not shown).

For both sexes and each cancer group, being born outwith Scotland was associated with reduced risk of cancer compared to being born in Scotland. The only exception was lung cancer risk for males (RR 0.90, 95% CI 0.81–1.00) (Tables 4 and 5). Regardless of cancer group or sex, being single was associated with reduced cancer risk compared to being married. For females, being divorced or widowed was associated with increased cancer risk compared to the reference regardless of cancer group. For males being divorced was associated with increased risk for lung and UADT cancer while being widowed was associated with increased lung cancer risk only (Tables 4 and 5).

**Table 4:** Tables 4. Fully adjusted^1^ relative risks (RR) and 95% confidence intervals (CI) by cancer, demographic or socioeconomic variable for females, Scotland 1991–2006.

		All cancer				Lung				UADT			
	Level	Number of cases	RR	95% CI		Number of cases	RR	95% CI		Number of cases	RR	95% CI	
Country of birth	Scotland	10946	Reference			1344	Reference			416	Reference		
	Rest UK	869	**0.90**	**0.85**	**0.95**	66	**0.66**	**0.61**	**0.72**	30	**0.86**	**0.79**	**0.93**
	Rest of World	254	**0.74**	**0.67**	**0.82**	20	**0.52**	**0.45**	**0.61**	7	**0.54**	**0.46**	**0.64**
Marital status	Married	6721	Reference			767	Reference			235	Reference		
	Divorced	696	**1.12**	**1.05**	**1.19**	92	**1.17**	**1.09**	**1.26**	34	**1.55**	**1.43**	**1.68**
	Single	1979	**0.91**	**0.87**	**0.95**	94	**0.60**	**0.55**	**0.64**	51	**0.88**	**0.82**	**0.95**
	Widowed	2673	**1.07**	**1.03**	**1.12**	477	**1.29**	**1.23**	**1.35**	133	**1.08**	**1.03**	**1.14**
Area deprivation	1 Least Deprived	899	Reference			65	Reference			31	Reference		
	2	993	0.93	0.87	1.00	88	1.06	0.95	1.18	34	**0.84**	**0.75**	**0.93**
	3	1359	0.95	0.89	1.02	119	0.97	0.87	1.07	49	**0.89**	**0.81**	**0.99**
	4	1502	1.01	0.95	1.08	155	**1.15**	**1.04**	**1.27**	46	**0.81**	**0.73**	**0.89**
	5	1324	0.95	0.89	1.02	158	**1.17**	**1.06**	**1.29**	37	**0.64**	**0.58**	**0.72**
	6	1347	0.97	0.91	1.04	151	1.05	0.95	1.17	56	0.95	0.86	1.05
	7	1217	0.99	0.93	1.07	147	1.11	1.00	1.23	48	0.97	0.88	1.07
	8	1204	1.00	0.93	1.07	155	**1.17**	**1.05**	**1.29**	49	0.97	0.87	1.07
	9	1100	0.97	0.90	1.05	195	**1.52**	**1.37**	**1.68**	52	1.07	0.96	1.18
	10 Most deprived	1124	1.00	0.93	1.08	197	**1.53**	**1.38**	**1.69**	51	1.09	0.98	1.21
Economic activity	Economically active	4126	Reference			310	Reference			109	Reference		
	Economically inactive	7864	**1.06**	**1.02**	**1.11**	1120	**1.29**	**1.22**	**1.36**	344	**1.20**	**1.12**	**1.28**
	Under 16 years old	79	0.93	0.77	1.12	0	0.00	0.00	0.00	0	0.00	0.00	0.00
Education level	First degree and higher	386	Reference			12	Reference			7	Reference		
	Other non degree	806	0.96	0.88	1.06	56	**1.57**	**1.27**	**1.94**	25	**1.46**	**1.21**	**1.75**
	None	9979	0.99	0.91	1.08	1280	**1.94**	**1.60**	**2.37**	394	**1.42**	**1.20**	**1.69**
	Under 18 years old or missing^2^	898	0.96	0.87	1.06	82	**1.66**	**1.35**	**2.05**	27	1.20	1.00	1.46
Occupational social class	I, II Professional, managerial, technical	1638	Reference			120	Reference			41	Reference		
	IIIa N Skilled non manual	2250	1.00	0.95	1.06	157	**0.83**	**0.76**	**0.90**	63	1.07	0.98	1.18
	IIIb M Skilled manual	525	1.03	0.95	1.12	65	**1.27**	**1.14**	**1.41**	21	**1.45**	**1.29**	**1.64**
	IV Partly skilled	1137	1.06	0.99	1.13	117	1.08	0.98	1.18	38	**1.33**	**1.20**	**1.48**
	V Unskilled	926	1.04	0.97	1.11	173	**1.36**	**1.25**	**1.48**	37	**1.22**	**1.10**	**1.36**
	No job in last 10 years, under 16 years old or missing^3^	5593	0.98	0.92	1.04	798	0.99	0.91	1.07	253	**1.15**	**1.05**	**1.26**
Car ownership	1 or more car(s)	6427	Reference			560	Reference			195	Reference		
	No car	5642	**1.07**	**1.03**	**1.11**	870	**1.27**	**1.21**	**1.33**	258	**1.23**	**1.16**	**1.29**
Household tenure	Owned	5987	Reference			515	Reference			200	Reference		
	Rented	6082	**1.08**	**1.04**	**1.11**	915	**1.34**	**1.28**	**1.40**	253	1.02	0.97	1.07

1 Fully adjusted model is adjusted for age and mutually adjusting all the variables for each other.

2 4.6% of total population was under 18 years old therefore education not recorded; 4.6% of total population education level not stated.

3 0.4% of total population occupational social class was not adequately described or not stated, 35.7% of total population was less than 16 years old or held no job in last 10 years.

Source: Scottish Longitudinal Study.

**Table 5:** Tables 5. Fully adjusted^1^ relative risks (RR) and 95% confidence intervals (CI) by cancer, demographic or socioeconomic variable for males, Scotland 1991–2006.

		All cancer				Lung				UADT			
	Level	Number of cases	RR	95% CI		Number of cases	RR	95% CI		Number of cases	RR	95% CI	
Country of birth	Scotland	8765	Reference			1894	Reference			698	Reference		
	Rest UK	731	**0.88**	**0.84**	**0.93**	125	**0.85**	**0.79**	**0.92**	47	**0.78**	**0.72**	**0.85**
	Rest of World	267	**0.87**	**0.80**	**0.94**	56	0.90	0.81	1.00	8	**0.39**	**0.32**	**0.47**
Marital status	Married	7452	Reference			1558	Reference			552	Reference		
	Divorced	449	1.05	0.98	1.13	123	**1.15**	**1.06**	**1.24**	55	**1.28**	**1.18**	**1.39**
	Single	941	**0.69**	**0.66**	**0.73**	140	**0.54**	**0.50**	**0.58**	92	**0.72**	**0.67**	**0.77**
	Widowed	921	0.98	0.94	1.04	254	**1.09**	**1.03**	**1.16**	54	0.99	0.91	1.07
Area deprivation	1 Least Deprived	790	Reference			92	Reference			46	Reference		
	2	819	0.93	0.87	1.00	130	**1.17**	**1.05**	**1.31**	46	**0.85**	**0.76**	**0.95**
	3	1124	0.98	0.92	1.04	187	**1.18**	**1.06**	**1.30**	90	**1.22**	**1.10**	**1.35**
	4	1183	0.96	0.90	1.02	208	**1.16**	**1.05**	**1.28**	93	**1.15**	**1.04**	**1.28**
	5	1116	1.00	0.94	1.07	229	**1.35**	**1.22**	**1.49**	87	**1.18**	**1.07**	**1.31**
	6	1043	0.97	0.91	1.04	226	**1.31**	**1.18**	**1.45**	71	0.90	0.81	1.01
	7	1002	1.05	0.98	1.13	237	**1.48**	**1.34**	**1.63**	88	**1.24**	**1.12**	**1.38**
	8	974	1.02	0.95	1.10	238	**1.45**	**1.31**	**1.60**	77	1.05	0.94	1.17
	9	874	1.04	0.97	1.12	249	**1.65**	**1.49**	**1.83**	86	**1.26**	**1.13**	**1.40**
	10 Most deprived	838	1.08	1.00	1.16	279	**1.89**	**1.71**	**2.10**	69	0.97	0.86	1.08
Economic activity	Economically active	4069	Reference			671	Reference			335	Reference		
	Economically inactive	5685	**1.14**	**1.10**	**1.19**	1404	**1.28**	**1.22**	**1.35**	418	**1.45**	**1.37**	**1.53**
	Under 16 years old	9	**0.38**	**0.24**	**0.60**	0	0.00	0.00	0.00	0	0.00	0.00	0.00
Education level	First degree and higher	481	Reference			39	Reference			26	Reference		
	Other non degree	534	0.98	0.90	1.06	59	**1.24**	**1.05**	**1.45**	36	1.10	0.95	1.26
	None	8298	1.06	0.99	1.13	1861	**1.95**	**1.70**	**2.22**	660	**1.14**	**1.01**	**1.28**
	Under 18 years old or missing^2^	450	0.91	0.83	1.00	116	**1.95**	**1.68**	**2.27**	31	0.92	0.79	1.07
Occupational social class	I, II Professional, managerial, technical	2001	Reference			287	Reference			120	Reference		
	IIIa N Skilled non manual	696	1.03	0.97	1.09	109	0.93	0.85	1.02	52	**1.28**	**1.17**	**1.40**
	IIIb M Skilled manual	2384	**1.06**	**1.01**	**1.11**	541	**1.19**	**1.12**	**1.27**	227	**1.47**	**1.37**	**1.58**
	IV Partly skilled	1394	**1.09**	**1.03**	**1.14**	291	1.07	1.00	1.15	119	**1.30**	**1.21**	**1.41**
	V Unskilled	421	1.08	1.00	1.17	97	1.10	1.00	1.21	49	**1.68**	**1.52**	**1.85**
	No job in last 10 years, under 16 years old or missing^3^	2867	1.04	0.99	1.10	750	**1.21**	**1.13**	**1.29**	186	1.23	1.14	1.34
Car ownership	1 or more car(s)	6150	Reference			1073	Reference			430	Reference		
	No car	3613	1.01	0.97	1.04	1002	**1.17**	**1.12**	**1.22**	323	**1.34**	**1.27**	**1.41**
Household tenure	Owned	5199	Reference			847	Reference			360	Reference		
	Rented	4564	1.01	0.97	1.04	1228	**1.23**	**1.17**	**1.28**	393	**1.10**	**1.05**	**1.15**

1 Fully adjusted model is adjusted for age and mutually adjusting all the variables for each other.

2 4.6% of total population was under 18 years old therefore education not recorded; 4.6% of total population education level not stated.

3 0.4% of total population occupational social class was not adequately described or not stated, 35.7% of total population was less than 16 years old or held no job in last 10 years.

Source: Scottish Longitudinal Study.

Regardless of sex, all cancer risk was not associated with area deprivation. For females, lung cancer RRs were more variable among those from more affluent area deprivation deciles, but showed clear increased risk association for the three most deprived deciles. For males and compared to females, lung cancer RRs for area deprivation were more pronounced showing clear increasing gradient of elevated risk for all area deprivation deciles. For females, area deprivation was associated with reduced UADT cancer for the more affluent deciles while the 95% CI for more deprived deciles included 1.0. For males and UADT cancer, RRs 95% CIs were generally greater than 1.0 suggesting association with stronger increased risk compared to females, but were more variable for the more deprived area deciles (Tables 4 and 5).

Regardless of sex or cancer group, increased cancer risk was associated with inactive economic status. For males, UADT cancer risk (RR 1.45, 95% CI 1.37–1.53) was strongest followed by lung and then all cancer. For females the cancer group order starting with the highest risk was lung cancer (RR 1.29, 95% CI 1.22–1.36), UADT then all cancer. For both males and females, education level was not associated with all cancer risk. Regardless of sex, no education or holding a non degree qualification was associated with increased lung cancer risk compared to holding a degree. For females, elevated UADT cancer risk was also associated with these categories; but only associated with no education for males (Tables 4 and 5).

For UADT cancer risk and compared to the professional, managerial and technical reference, most occupational social class categories were associated with increased RRs for both males and females. Occupational social class associations with lung cancer risk were very limited (males) or variable (females) while all cancer risk were limited (males) or did not exist (females). Having no access to a car was associated with increased risk compared to owning a car regardless of cancer group and sex with the exception of all cancer risk in males. Renting a home was associated with increased lung cancer risk compared to owning a home for both sexes. Likewise elevated UADT cancer risk was associated with home rental for males, but not females while elevated all cancer risk was associated with home rental for females but not males (Tables 4 and 5).

For males, highest qualification (lung), social class (all cancer, lung), car ownership (lung, UADT), and housing tenure (lung, UADT) presented statistically significant interactions with area, while for females, social class (lung), housing tenure (lung, UADT) and car ownership (UADT) interactions with area were statistically significant (P<0.0001, data not shown). Exploratory sub group analysis of the statistically significant interactions uncovered no discernable trends as even a single cross-product category can trigger significance (Data not shown).

Regardless of sex and cancer group, elevated risk was associated with no education and living in deprived areas. RRs for males exceeded those for females and risk order was consistent for both sexes (lung followed by UADT with all cancer the lowest elevated risk). For males, elevated risk was associated with all area-education level combinations regardless of cancer group excluding the all cancer risk among males with a degree living in deprived areas. Elevated lung cancer risk in females was also associated with no education living in more affluent areas (RR 1.77, 95% CI 1.22–2.36) (Table 6).

**Table 6:** Area deprivation and education interrelationship: age adjusted relative risks (RR) and 95% confidence intervals (CI) by cancer and sex, Scotland 1991–2006.

		Female			Males		
		RR	95% CI		RR	95% CI	
All cancer	Deprived area: no education	**1.13**	**1.06**	**1.22**	**1.21**	**1.12**	**1.30**
	Deprived area: diploma or higher education	0.93	0.81	1.07	1.01	0.85	1.19
	Affluent area: no education	1.05	0.98	1.12	**1.12**	**1.04**	**1.21**
	Affluent area: diploma or higher education	Reference			Reference		
Lung cancer	Deprived area: no education	**2.62**	**1.97**	**3.49**	**3.65**	**2.87**	**4.63**
	Deprived area: diploma or higher education	1.27	0.74	2.20	**2.04**	**1.31**	**3.20**
	Affluent area: no education	**1.77**	**1.33**	**2.36**	**2.36**	**1.85**	**3.00**
	Affluent area: diploma or higher education	Reference			Reference		
UADT cancer	Deprived area: no education	**1.64**	**1.09**	**2.49**	**2.10**	**1.55**	**2.84**
	Deprived area: diploma or higher education	1.18	0.53	2.61	**1.80**	**1.02**	**3.18**
	Affluent area: no education	1.21	0.80	1.85	**1.75**	**1.29**	**2.38**
	Affluent area: diploma or higher education	Reference			Reference		

Source: Scottish Longitudinal Study.

## Discussion

We found a complex and different pattern of socioeconomic factors associated with risk in different cancer groups in both sexes with no single factor predominant.

Being born in Scotland was associated with increased risk regardless of the cancer group and sex and is well established in the literature [20]; [21]. The observed lack of any difference for lung cancer risk in males compared to the rest of the world may reflect the different stage in the smoking epidemic in Scotland for males and females relative to each other as well as the transfer of the epidemic from the developed to the developing world [32]–[34].

Relative to being married and in contrast to females, we found no all cancer or UADT cancer risk differences for widowed males but not for widowed females. This may reflect financial implications of widowhood for a cohort of older women where marriage imparted greater financial security and little or no change in financial security for their male counter parts. We also found being divorced or widowed was associated with increased cancer risk for females while being single was associated with reduced risk for both sexes. Our results are broadly consistent with Danish studies identifying increased lung [35], mouth and pharyngeal [4], and laryngeal [4] cancer risk associated with being divorced or widowed for both sexes. In contrast to our results for UADT cancer, being single was associated with elevated head and neck cancer risk in two Danish studies and one Italian [4]; [35]; [36]. The Danish studies separately identified cohabiting and single individuals while our study was limited to legal marriage categories only. Reduced risk levels for single individuals seen in our study may reflect the risk of individuals who were cohabiting but legally single as well as the risk of single individuals living alone. Many have suggested cohabiting or married individuals experience improved health status due to stronger social relationships and potentially healthier behaviours reflecting greater psychological reinforcement provided by partner support, while being divorced or widowed may increase unhealthy behaviour due to reduced income and increased stress [36]–[38]. Poverty and social exclusion also has the effect of increased risk of divorce and separation as well as disability, illness, addiction and social isolation [39].

Our finding that area deprivation remained significant for lung cancer risk even after adjustment for the individual SES factors is consistent with others who found increased neighbourhood population density and unemployment were associated with increased lung cancer risk [40]. This neighbourhood effect of increased risk may reflect physical and social environment e.g., exposure to traffic or industrial related air pollution, reduced access to shops and services promoting healthier lifestyles and increased stressful environments and general sense of hopelessness associated with lack of supportive social networks, resources and opportunity [41]; [42]. In the context of area air quality, a recent review of several European and US studies focusing on air pollution and the respiratory system found between 7 – 30% of lung cancer incidence was attributed to chronic exposure to air pollution [43]. Consistent with other parts of the UK, in Scotland, greater air pollution concentrations were found in the more deprived deciles reflecting heavier road traffic in cities and higher proportion of deprived populations in urban locations. When compared to England and Northern Ireland, however, the inequality gradient associated with air pollution concentration was less steep in Scotland [44]. Relative to the rest of the UK, higher lung cancer incidence rates in Scotland in general and among the more deprived areas does not appear to reflect current higher air pollution levels. Nevertheless and despite being below WHO guidelines [45], air pollution in Scotland is greatest in more deprived areas. This may contribute to an already ‘unhealthy’ neighbourhood environment in deprived areas adding to stress and exacerbating already unhealthy lifestyles which potentially lead to lung cancer diagnosis or diagnosis at an even earlier age among the more deprived [10].

Similar to our results, several studies have reported that not working versus working was associated with elevated risk of all cancer [19], lung [35] mouth and pharyngeal [4], laryngeal [4], oesophageal cancers [16] and oral cancer [5] for both sexes. Unemployment and negative health consequences are well established with health effects felt at the first signs of job insecurity leading to psychological stress and anxiety as well as financial impact [39]; [46].

Our findings in relation to the elevated cancer risk associated with no education are largely consistent with others who found reduced mouth and pharyngeal cancer risk for males with higher education attainment and no risk difference for females for these cancers [4]. No risk differences were also previously reported for education attainment and oesophageal cancer for both sexes [16], while reduced lung cancer risk associated with higher education attainment was found for both sexes [35]. In relation to the role of early years on the life course, education is recognised as a key factor in establishing a foundation for adult life, and many studies suggest that education inequalities may have an underpinning role in health and social inequalities influencing the occupation attained and income earned in later life [2]; [26]. While we have not been able to establish education as the most important factor influencing health outcome, others studying the impact of socioeconomic circumstances on health (including cancer incidence) over the life course concluded that education level is the primary determinant [47]. Our results may be explained by the theory that the relative importance of education may be dependent on levels of other SES measures suggesting that education was less important to health status among individuals who reside in households below poverty thresholds [48].

After full adjustment, our finding of increased UADT cancer risk for most occupational social class categories compared to the professional, managerial and technical group in both males and females is consistent with others studying mouth, pharyngeal and laryngeal cancer [4]. However, oesophageal cancer risk for females has previously not been associated with social class [16]. Although the number of cases in our study did not allow disaggregation of UADT cancers, this is consistent with our previous findings of differences in SES association with oesophageal cancer risk between the sexes (females weaker than males) as well as differences in SES association with different oesophageal cancer morphologies (increased risk association for squamous cell carcinoma and no association for adenocarcinoma) [10]. However our previous study did not explore any individual socioeconomic variables, including occupational social class. Furthermore, oropharyngeal cancer, ranked relatively low in terms of contribution to socioeconomic inequalities of all cancer risk for both males and females is one of the fastest increasing cancers in Scotland [10]. However and in contrast to others, for the present study we did not find a strong association with lung cancer risk in either sex [35]. This may reflect the higher proportion of individuals who were economically inactive or had not held a job in the last 10 years (Tables 4 and 5). Our findings of occupational social class association with increased cancer risk is likely to reflect not only employment status but also prestige, qualifications, rewards, and job characteristics (e.g. reporting relationship, locus of control and autonomy) all of which have been associated with social status differences in health, sickness absence and premature death [39]. Having a job is better for health outcomes than being unemployed, but the nature of the social relationships and their implication for stress at work can negatively contribute to illness [49]. The stronger increased UADT cancer risk association we found for males compared to females is consistent with the theory that the socioeconomic roles performed by males and females differ. For women, health is more negatively affected by the psychosocial stress over the life course of balancing caring, paid work and managing a household while work conditions alone more frequently negatively affect men's health [50].

Compared to owning a car we found that no car access was associated with increased risk of all cancer groups for females, but only for lung and UADT cancer for males. Our observation that no car access was not associated with increased risk for all cancer in males is likely to reflect the mix of cancer sites included in this cancer group, some of which are more likely to be diagnosed among more affluent individuals (e.g. prostate cancer and melanoma) who are more likely to be car owners while other cancers are more likely to be diagnosed among the more deprived (lung and UADT cancer) who are less likely to own a car. Consistent with our results where lack of car access is associated with elevated lung cancer risk, Lancaster et al. established elevated risk association regardless of sex in North England [51]. The 2011 Scottish Household Survey (SHS) indicated car availability was strongly associated with income and car access differed by sex with 76% of males and only 60% of females holding a license [52]. In our study, the proportion of car owners by sex for the full cohort is consistent with the SHS results (Table 1). The higher lung cancer RR for women without a car compared to men may reflect differences in the smoking epidemic stage between men and women as well as the general shift in prevalence of the smoking habit from the more affluent to the more deprived as the more affluent adopt healthier non-smoking behaviour more quickly. The lower UADT RRs for women without a car compared to men is likely to reflect the weaker association of deprivation with UADT cancer risk among women. Our results suggest for both sexes, to a lesser or greater degree depending on sex and cancer, car ownership as a marker of material wealth and as a resource enabling access to work, schools, shops, leisure activities, friends and family, is an important socioeconomic dimension associated with cancer risk [53].

Several Danish studies established increased risk associated with rented compared to owner occupied accommodation for all cancer [19], lung [35], mouth and pharynx [4], laryngeal [4] and oesophageal [16] cancer regardless of sex. In contrast, we found this was not the case for women and UADT cancer risk or for men and all cancer risk. With respect to women diagnosed with UADT cancer, we expected renting to be associated with higher risk compared to the home owner category as housing condition is independently associated with deterioration of health, especially in women. Furthermore, renters are more likely to report more housing problems than owner occupiers [53]. The differences may reflect that household tenure is a material wealth indicator and the finding that deteriorating health applies to women home owners in poverty as well as renters [29]; [54]. Finally, these results may reflect the weaker association of UADT cancer with socioeconomic status for women compared to men [10]. Like the results for no car access, no difference in all cancer risk for males is likely to reflect the mix of cancer sites included in the all cancer group some of which are more likely to be diagnosed in the more affluent while other cancers are more likely to be diagnosed among the more deprived.

Our findings on the inter-relationship between area deprivation and education show the synergistic effect of area and individual SES measured by education and are consistent with others focusing on cancer [22] and lung function [40]. Consistent with others we too found, low education level and high deprivation was associated with increased lung and UADT cancer risk in males and the risk order implied greater influence of education [22]. For females, being educated to some extent mitigated the effects of living in a deprived area; likewise living in an affluent area mitigated the effect of no education. Given these cancers are largely driven by smoking and alcohol behaviours, which are both more prevalent among the more deprived [10] implies that social and cultural aspects of SES are important in uptake and continuation of smoking and alcohol consumption [22]. Education level captures the impact of socioeconomic and cultural circumstances at an early age when adopting the habit. In addition, the differences between the sexes in the smoking epidemic are likely to explain the mitigating effects identified.

It has been suggested that low SES, regardless of measure, potentially implies some form of ‘stress’ which may come from a range of sources e.g., insecurity of work, unemployment, fear of crime, debt, low material resources and low social capital and community cohesion [39]; [46]. Lifelong adverse experiences have strong and long lasting deleterious effects on health and occur most often among the most deprived [55]. Furthermore disadvantage at critical life transition points such as early childhood, moving from primary to secondary school, starting work, leaving or moving home, starting a family, job change, facing redundancy and retirement are also known to contribute to deteriorating health status [39]. Recent studies report telomere lengths which vary by age, sex and ethnicity are associated with biological ageing and cancer [65]. Various studies have explored the predictive potential of telomere length for cancer risk [56]–[58] and its association with different socioeconomic variables [59]; [60] such as low relative household income, renting a home and life style factors including poor diet [59] or adverse early life experiences [61]. Cancers strongly associated with smoking such as lung cancer display most consistent results showing shorter telomere length association with incidence [62]. Behaviours such as smoking [62], alcohol consumption abuse [63], and obesity [62] are also associated with accelerated telomere attrition as well as recognised as risk factors for lung and UADT cancer which are associated with lower socioeconomic circumstances in Scotland [10].

To date, many studies have focused on cancer mortality; here for the first time in Scotland, we use multiple individual SES metrics as well as an area measure to explore cancer risk. Area rather than individual measures of SES, created for the smallest available administrative unit, out of necessity, are increasingly used world wide to measure effects of SES on health outcomes and to plan services [7] and may be used as surrogates for individual social indicators [64]–[68]. Our study recognises that individual SES classification based on area SES measures may not reflect individual SES accurately (‘ecological fallacy’) [69]; [70] as well as the importance of investigating the influence of individual as well as area socioeconomic circumstances when considering SES as the exposure [26]. We also present for the first time linkage of SCR incidence data with the SLS providing a large cohort and number of primary tumours followed for several years. Finally, the SCR is a population based cancer registry with evidence of high data quality and less than 1% of cases identified through death certification only [71].

We excluded any diagnosis of cancer prior to the April 1991 Census and cohort start; this coupled with measurement of area and individual SES variables at the 1991 Census provided measurement at the earliest time possible prior to diagnosis. This gives us the advantage of knowing individual SES before cancer diagnosis rather than the traditional area measurement at time of diagnosis. Measurement at time of diagnosis may reflect the reverse impact of diagnosis on socioeconomic circumstances. Therefore our finding suggesting a strong role of low SES is notable. Furthermore, given all variables used to establish the area deprivation measure were included in our model (excluding accommodation overcrowding due to no discernable differences in the cohort population), the fact that that these variables remained statistically significant in the fully adjusted model further supports the argument of a separate and independent role of individual socioeconomic factors in addition to the area measure. However, these results may reflect confounding by other unavailable and unmeasured factors including geographic attributes such as environmental pollution, individual risk behaviour and other individual SES variables such as an income metric and house value, a potentially important individual SES measure given the cancers under investigation are most likely to be diagnosed among the old who are also more likely to have access to accumulated wealth.

To capture socioeconomic circumstances at the earliest point in the study we used the Registrar General's occupational social class [72]. However, this measure focuses on manual versus non manual distinction between occupations and is only applicable to those in paid employment, omitting important segments of society such as the unemployed, retired and permanently sick [72]–[74]. Finally, as indicated previously, we did not have access to any risk behaviour data.

We have used person-years models in our analysis which estimate the risk of cancer incidence in the absence of competing risks, even those competing risks that may be correlated (for example, a smoking related cause of death other than cancer). Because individuals succumbing to a non cancer smoking-related death may be at greater risk of cancer had they lived, the estimated risks may understate the effects of the variables under investigation. However, because we desire to measure the association of SES exposure with cancer incidence, in effect performing a prognostic marker effect test, this approach is preferred to alternatives such as the cumulative incidence function [75]. It may be suggested that multi-level modelling would have been a more suitable analytical approach given we are exploring one area and five individual SES indicators. Our only area deprivation indicator (Carstairs) is measured at postcode sector level of which there are 1011 in Scotland. Given the small number of cases by cancer group and sex, there were many postcode sectors with either no or only a very few cases and therefore no individual measurements available. As a result, multi-level modelling was not appropriate for our data. Finally, the approach adopted (fully adjusted model) recognises our a priori hypothesis (and conscious SES variable selection) that different individual SES variables capture different SES dimensions at different points in the life course. Area measures of socioeconomic inequality, including the one used in this study are frequently composite measures reflecting a number of different aspects of socioeconomic circumstances. For area deprivation measures, a composite index is often used to capture as much of the multi-dimensional nature of deprivation as possible. In our study, depending on the cancer and sex, both the area measure and the included individual variables were associated with cancer risk to various magnitudes. This complex picture is likely to be further complicated by other unavailable demographic or socioeconomic dimensions (such as ethnicity [49], long term income [76] and wealth [77]). Despite this emerging understanding, for cancer risk, few, if any composite individual measures tailored to the specific population and outcomes have been considered.

## Conclusion

Our study recognises the strengths and weaknesses of relying on area measures of deprivation alone and begins to reassess more finely the socioeconomic factors associated with cancer risk.

This association of multiple socioeconomic and demographic variables with cancer risk is likely to reflect not only the complex, multifaceted nature of deprivation, but also the various and cumulative effects of different socioeconomic determinants over the life course and between generations [78] which in themselves reflect the fact that an individual's socioeconomic circumstances may change over the course of their life, the impact of which can accumulate over time. This complexity is also likely to reflect the longer lag time between exposure and diagnosis for cancer incidence; for example, lung cancer lag period is estimated at several decades [79].

We identified that different socioeconomic variables are not proxies of each other, but are independently associated with different cancer risks in both sexes. No single measure of socioeconomic circumstances comprehensively reflects all aspects of socioeconomic stratification or captures the full effect of low socioeconomic circumstances at different stages in the life course or transmitted over generations. The different components of SES not only suggest different cohort subgroups, but point to different pathways such as different behaviours or to critical periods of the life course. Our results emphasize the importance of using multiple SES measures in epidemiological studies.

In conclusion, different and independent socioeconomic variables are inversely associated with different cancer risks in both sexes; no one socioeconomic variable on its own captures all aspects of socioeconomic circumstances or life course. Association of multiple socioeconomic variables is likely to reflect the complexity and multifaceted nature of deprivation as well as the various roles of these dimensions over the life course which in turn reflects the longer gestation period for cancer.
